# Carbon black nanoparticles induce cell necrosis through lysosomal membrane permeabilization and cause subsequent inflammatory response

**DOI:** 10.7150/thno.34065

**Published:** 2020-03-15

**Authors:** Xia Yuan, Wen Nie, Zhiyao He, Jingyun Yang, Bin Shao, Xuelei Ma, Xiangxian Zhang, Zhenfei Bi, Lu Sun, Xiao Liang, Yan Tie, Yu Liu, Fei Mo, Dan Xie, Yuquan Wei, Xiawei Wei

**Affiliations:** Laboratory of Aging Research and Cancer Drug Target, State Key Laboratory of Biotherapy and Cancer Center, National Clinical Research Center for Geriatrics, West China Hospital, Sichuan University, No. 17, Block 3, Southern Renmin Road, Chengdu, Sichuan 610041, PR China.

**Keywords:** Carbon black nanoparticles, Macrophages, Necrosis, Mitochondrial DNA, Inflammation

## Abstract

**Rationale**: The adverse health effects of nano-particulate pollutants have attracted much attention in recent years. Carbon nanomaterials are recognized as risk factors for prolonged inflammatory responses and diffuse alveolar injury. Previous research indicated a central role of alveolar macrophages in the pathogenesis of particle-related lung disease, but the underlying mechanism remains largely unknown.

**Methods**: C57BL/6 mice were intratracheally instilled with carbon black nanoparticles (CBNPs). Cell necrosis and the infiltrated neutrophils in the lungs were detected by flow cytometry. Release of mitochondria was observed with Mito Tracker and mitochondrial DNA (mtDNA) was quantified by qPCR via Taqman probes. TLR9-p38 MAPK signaling pathway was detected by Western blotting. The production of lipid chemoattractant leukotriene B4 (LTB4) in the supernatant and bronchoalveolar lavage fluid (BALF) was quantitated using an enzyme immunoassay (EIA).

**Results**: In the present study, we found that a single instillation of CBNPs induced neutrophil influx in C57BL/6 mice as early as 4 h post-exposure following the rapid appearance of cell damage indicators in BALF at 30 min. Macrophages exposed to CBNPs showed necrotic features and were characterized by lysosome rupture, cathepsin B release, reactive oxygen species generation, and reduced intracellular ATP level. Necrosis was partly inhibited by a specific lysosomal cathepsin B inhibitor CA074 Me. Further analyses suggested that the resulting leakage of mtDNA from the necrotic cells activated neutrophils and triggered severe inflammation *in vivo*. Pulmonary neutrophilic inflammation induced by mtDNA was reduced in TLR9^-/-^ mice. Additionally, mtDNA induced LTB4 production from macrophages, which may contribute to neutrophil recruitment.

**Conclusion**: We demonstrated here that CBNPs induce acute cell necrosis through lysosomal rupture and that mtDNA released from necrotic cells functions as a key event mediating pulmonary neutrophilic inflammation. This study described a novel aspect of the pathogenesis of particle-induced inflammatory response and provided a possible therapeutic target for the regulation of inflammation.

## Introduction

In recent decades, nanotechnology has rapidly developed and nanomaterials present unique properties that facilitate applications in electronics, engineering and biomedicine 1. Meantime, nanoparticles (NPs) as novel carriers provide a promising strategy for improving the effectiveness of immunotherapy and anticancer therapies 2-4. Carbon nanomaterials are highly promising nanomaterials, with different formations such as single-walled nanotubes (SWCNTs), multi-walled carbon nanotubes (MWCNTs), carbon black nanoparticles (CBNPs), fibers, and fullerenes 5-10. Particulate matter (PM)-induced air pollution is a significant public health concern, especially pollution from ultrafine particles (≤ 0.1 µm) 11. Nano-sized PMs generate greater threats to public health due to the great surface area and reactivity. CBNPs are a major part of particulate ambient pollution and can cause pulmonary toxicity mainly through inflammation 12,13.

CBNPs are widely used in rubber, plastics, inks, and paints, 14 and occupational exposure dramatically increases with mass production of CBNPs. During production, NPs are inadvertently released into the atmosphere, which poses inhalation hazard for general population 15,16. In addition to manufactured CBNPs, there exist naturally occurring nanoscale carbon particles. The main source of ultrafine particles is diesel exhaust, and carbonaceous nanoparticles are an important part of diesel exhaust 17,18. Combustion of fuel and waste generates large amounts of CBNPs in daily life 19. Daily exposure may occur from ultrafine ambient nanoparticles in the environment or from consumable products. Insoluble CBNPs are inhaled and induce pulmonary toxicity 20,21.

In lungs, alveolar macrophages maintain the first line of defense by phagocytosing and clearing foreign materials 15. CBNPs exist in many different environmental exposures, and an immune response can be generated by alveolar macrophages following the phagocytosis of deposited particles within the respiratory tract 22 via release of inflammatory cytokines and in some cases cell death 23.

Necrosis is an accidental and proinflammatory form of cell death characterized by a rapid loss of plasma membrane integrity, leading to the release of endogenous molecules called damage-associated molecular patterns (DAMPs) 24-27. Sterile inflammation occurs as response to cell necrosis in several diseases, and endogenous danger signals released from necrotic cells contribute to profound inflammatory responses 28,29. Mitochondrial DNA (mtDNA) shares many features similar to that of its bacterial ancestors and consists of a circular genome, containing abundant unmethylated DNA known as CpG islands 30,31. The mtDNA release following cellular damage and lung injury reportedly functions as a DAMP, contributing to lung inflammation 31,32.

The health effects of nano-particulate pollutants have raised social concerns. CBNPs are risk factors for diffuse alveolar injury and prolonged inflammatory responses 20,23, but the underlying mechanism remains largely unknown. In this study, our aim was to identify the potentially important molecular events of CBNP-induced lung inflammation, and to clarify the relationship between lung inflammation and cell necrosis induced by CBNPs. We postulated that CBNPs were phagocytosed by alveolar macrophages, causing acute cell necrosis. The released mtDNA from necrotic cells recruits and activates inflammatory cells.

## Materials and methods

### Animals

All animals were of C57BL/6 background. For experiments, animals were matched in sex and age that is female, 6-8 weeks. TLR9^-/-^ mice with C57BL/6 background were obtained from Bioindustry Division Oriental Yeast Co., Ltd. (Tokyo, Japan). Female C57BL/6 WT mice were purchased from Vital River (Beijing, China). All animals were maintained under specific pathogen-free conditions. All animal experiments were performed according to the guidelines of the Institutional Animal Care and Use Committee of Sichuan University (Chengdu, Sichuan, China), and the protocols were approved by the Institutional Animal Care and Use Committee of Sichuan University (Chengdu, Sichuan, China).

### Preparation of instillation suspension

CBNPs were obtained from Sigma and stored in sterile and dry conditions. Before preparation of a suspension, CBNPs were baked at 200°C for 3 h to be made endotoxin-free. In the suspension, 4 mg of CBNPs was suspended in 1 ml of normal saline (NS) and the particle suspension (4.0 mg/ml) was de-agglomerated with the ultrasonic water bath for 30 min and the sample was vortexed every 10 min during the sonication procedure. The 4 mg/ml suspension was used for the high dose (0.2 mg/mice, 50 µl) and then diluted 1:20 in NS for the low dose (0.01 mg/mice).

### Characterization of instillation suspension

The hydrodynamic size and polydispersity of CBNPs were measured using a Mastersizer 2000 Laser Particle Size Analyzer (Malvern, British) at 25°C. Polydispersity values were utilized to evaluate the distribution of the nanoparticle population. The characteristics of the particles and the level of agglomeration were also evaluated by transmission electron microscope (TEM, H-6009IV, Hitachi, Japan). The Limulus Amoebocyte Lysate (LAL) gel-clot assay was used for endotoxin detection with a sensitivity of 0.25 EU/mL.

### Exposure of mice

CBNPs (0.01 mg or 0.2 mg) were administered to mice by a single intratracheal instillation. Each animal in the experimental group received a 50 µl suspension, and the control animals received an equal volume of vehicle (NS). Before surgery, the mice were anesthetized by ether inhalation. During the instillation procedure, the mice were placed on their backs on a 45 degree of slope. Hair in the central neck was shaved and the skin was disinfected with iodine. The trachea was gently exposed and intubated using a 29 gauge BD Insulin Syringe from the lower edge of cricoid cartilage, followed by an immediate up-right for 15 seconds. Then the mice were transferred to a 37°C heating plate until recovery from anesthesia.

### H&E staining and esterase staining

Lung tissues were fixed in 4% formalin for 72 h, embedded in paraffin and sectioned. Three-micrometer lung sections were stained with haematoxylin-eosin (H&E) following the manufacturer's instruction. Esterase staining was performed with Naphthol AS-D Chloroacetate Kit (Sigma) following the manufacturer's instruction. Stained slides were scanned with an upright microscope (Nikon) and the esterase-positive cells were counted in high-power fields (HPFs; 400×).

### Preparation of BALF cells and supernatant

Bronchoalveolar lavage fluid (BALF) was extracted three times with 1.0 mL of precooled 0.9% NS through the trachea. The BALF was stored on ice and were centrifugated at 4°C, 400 g for 10 min. The cell pellet and supernatant were kept separately for subsequent experiments.

### Neutrophil detection by flow cytometry

Mice were sacrificed and lung tissues were removed completely, cut up into small pieces and digested in type Ⅰ collagenase (1mg/ml, Gibco) at 37°C for 1-1.5 h with gentle vortex. Single-cell suspensions were obtained, washed twice, resuspended at a concentration of 10^6^ cells/ml and then stained with fluorochrome-conjugated anti-Ly-6G (BD Biosciences), anti-CD45 (BioLegend) and anti-CD11b (BioLegend) antibodies. For the detection of neutrophils in BALF, the cell pellet was resuspended in PBS and then stained with antibodies. Data were acquired on a NovoCyte flow cytometer (ACEA Biosciences) and analysis was carried out on NovoExpress software.

### PI/Annexin V detection by flow cytometry

For *in vitro* assay, cultured cells were harvested at 60-70% confluence and washed twice with cold PBS. For *in vivo* assay, BALF cells were harvested on ice, washed twice, and dispersed in cold PBS. Cells were double stained with PI/Annexin V (BD Biosciences) according to the manufacturer's instructions.

### LDH release detection

For *in vivo* experiments, BALF was extracted and centrifuged, and the supernatant was put on ice for immediate detection. For *in vitro* experiments, MH-S cells were cultured in 96-well dishes and treated with CBNPs (100 µg/cm^2^) or vehicle medium for 2 h, then centrifugated at room temperature, at 400×g for 10 min, and the supernatants were collected. The LDH levels in supernatants were detected with the LDH Cytotoxicity Assay Kit (Beyotime, China) following the manufacturer's protocol.

### Lysotracker-red staining

Lysotracker-red, a lysosomotropic probe (Beyotime, China) was used to label and track the acidic intracellular compartments (lysosomes) in live cells. The treated cells were incubated for 30 min at 37°C with Lysotracker-red (5 nM) and then washed three times. The fluorescence intensity was measured by flow cytometry.

### Dextran staining

MH-S cells were pre-loaded with 20-kDa FITC-conjugated dextran (Sigma) in warm RPMI-1640 complete medium at a concentration of 1mg/ml in the dark at 37°C, and 5% CO_2_ for 60 min. The cells were then treated with CBNPs (25 µg/cm^2^) or vehicle medium for 2 h.

### Cathepsin B staining

After treatment with CBNPs, alveolar macrophages were incubated with mouse anti-mouse cathepsin B antibody (Abcam, 1:400) and stained with FITC-goat anti-mouse secondary antibody for immunofluorescence.

### Colocalization staining of Mito Tracker and 8-OHdG

Alveolar macrophages were stained with Mito Tracker Red (Life Technology) and then treated with CBNPs (25 µg/cm^2^) or vehicle medium for 2 h. The cells were incubated with goat anti-mouse 8-OHdG antibody (Abcam, 1:300) and then stained with FITC-donkey anti-goat secondary antibody for immunofluorescence.

Data were acquired using a Leica TCS SP5 confocal microscope and were analyzed using LAS AF Lite.

### ROS determination

MH-S cells were pre-loaded with H2DCF-DA for 30 min and then treated with CBNPs (100 µg/cm^2^) or vehicle medium for 30 min. The fluorescence intensity of H2DCF-DA was measured by flow cytometry.

### Mitochondrial membrane potential assay

As previously described 33, Tetramethylrhodamine methyl ester (TMRM, Life Technology) was used as an indicator of mitochondrial membrane potential. After treated with CBNPs (100 µg/cm^2^) for 30 min, MH-S cells were harvested, washed twice, suspended at a density of 10^5^/ml and stained with TMRM at a final concentration of 20 nM for 30 min at room temperature. The cells were then washed three times with PBS and analysis was carried out with flow cytometry (as described above).

### ATP content detection

MH-S cells were treated with CBNPs (100 µg/cm^2^) or vehicle medium for 30 min and washed twice with cold PBS. ATP was measured using an ATP Assay kit (Abcam) and all procedures were conducted following the manufacturer's instructions. Each sample was replicated in three wells and the absorbance was measured using a microplate reader (Bio-Rad) at 570 nm.

### PI staining *in vivo* and *in vitro*

*In vivo*, mice were treated with CBNPs by intratracheal instillation. Four hours after administration, 0.5 µl PI (Sigma, 1mg/ml) was injected into the tail vein and 10-15 minutes later the mice were perfused with 4% formaldehyde through the tail vein. Then, lung tissues were removed completely, immediately snap-frozen, and 6-µm frozen sections were observed for PI-positive cells. *In vitro*, the treated cells in 96-well plates were incubated with 5 µl PI (BD Biosciences) in 100 µl medium for 5 min at room temperature and then representative images were photographed.

### Mito tracker staining

To observe the release of mitochondria caused by CBNPs, alveolar macrophages were stained with Mito Tracker at a final concentration of 50 nM for 30 min. Then, the cells were treated with CBNPs (25 µg/cm^2^) or vehicle medium for another 2 h. Finally, cells were fixed and stained with DAPI.

### Quantitative real-time PCR for mtDNA

For *in vivo* experiment, 4 h after instillation, BALF supernatants were collected. For *in vitro* experiment, MH-S cells were treated with CBNPs (100 µg/cm^2^) or vehicle medium for 4 h and the supernatants were collected. DNA in the supernatants was extracted with the QIAamp DNA Blood Mini Kit (Qiagen). Taqman probes were used for mtDNA quantification. Sequences of primers and probes were synthesized by Invitrogen. Sense primer, 5'-ACCTACCCTATCACTCACACTAGCA-3', antisense primer, 5'- GAGGCTCATCCTGATCATAGAATG-3', FAM-labeled TAMRA-quenched probes, 5'-ATGAGTTCCCCTACCAATACCACACCC-3'. The standard curve was constructed by serial dilutions of plasmid DNA containing the target PCR product.

### Preparation of mitochondria and mtDNA

Mouse lung tissues were isolated under sterile condition, placed on ice, and then homogenized using a mechanical pestle homogenizer. Mitochondria isolation kit (Qiagen) was used to prepare mitochondria, and mtDNA was extracted with mtDNA isolation kit (Abcam). All procedures were conducted on ice following the manufacturer's instructions. Protein concentration was determined with BCA (Thermo) after mitochondria was lysed. Determination of mtDNA was carried out on Nanodrop (Thermo) and no protein contamination was found. Mitochondria were kept at -80°C and mtDNA at -20°C for long-term storage.

### Isolation of bone marrow-derived macrophages, mouse bone marrow neutrophils, mouse peritoneal macrophages and alveolar macrophages

Mouse bone marrow-derived macrophages (BMDMs) were isolated as previously described 34. Briefly, BMDMs were prepared from 6-8-week-old mice (C57BL/6) and bone marrow cells were flushed into RPMI-1640, and cultured in complete RPMI-1640 medium supplemented with 20 ng/ml recombinant mouse macrophage colony-stimulating factor (M-CSF, BioLegend). Three days later, the medium was replaced with 10 ml of fresh growth medium and another 3 days later, cells were treated with mtDNA.

Mouse bone marrow neutrophils were isolated as previously described 35. Briefly, femurs and tibias were cut out in RPMI-1640 and bone marrow cells were flushed into 10 cm dishes. Cell suspensions were separated by density gradient centrifugation with Histopaque 1119 and Histopaque 1077 (Sigma-Aldrich) and the polymorphonuclear neutrophil (PMN) cell layer was harvested.

Mouse peritoneal macrophages were isolated by injecting 10 ml cold sterile NS into peritoneal cavity, followed by gentle massaging of the mouse abdomen for 10-15 s. Then the peritoneal fluid was collected slowly with a 19-gauge needle. The fluid containing the peritoneal cells was pooled together, resuspended in DMEM complete medium, and incubated at 37°C for 4 h.

Mouse alveolar macrophages were isolated as previously described 36. Briefly, BALF was collected and pooled under sterile conditions. Then, harvested cells were resuspended in DMEM complete medium, and after incubation at 37°C for 4 h, the non-adherent cells were discarded.

### Myeloperoxidase production and elastase release

Mouse bone marrow neutrophils were seeded in 24-well plate with RPMI complete medium at a concentration of 5 × 10^6^/ml. Then, mitochondria (200 µg/ml), mtDNA (2.5 µg/ml) and fMLF (1 µM) were added to the cells, and incubated at 37°C for 2 h. Activated neutrophils released myeloperoxidase (MPO) and elastase into medium. The medium was collected, centrifuged and the supernatants were kept in -80°C. MPO production and elastase release were determined using EnzChek Myeloperoxidase Activity Assay Kit (Invitrogen) and Mouse neutrophil elastase ELISA Kit (CUSABIO Life science, China), respectively.

### Chemotaxis assay

Chemotaxis assay was performed in transwells with 3 µm polyhydrocarbon filters (Corning). The fMLF (1 µM) and mtDNA (2.5 µg/ml) were incubated with cells for 4 h. The migrated neutrophils were counted in five randomly chosen HPFs (400×) following dehydration, fixation, and hematoxylin staining.

### Western blot assay

After incubation with indicated reagents for 2 h at 37°C, neutrophil culture medium (1ml) was collected and centrifuged. Protein samples of the residual pellet were prepared and protein concentration was determined by BCA (Thermo). Phospho-p38 MAPK (Cell Signaling) and p38 MAPK (Cell Signaling) were used as antibodies for Western blot assay.

### Inhibitor pretreatment

For ODN2088 (Invitrogen), *in vivo* ODN2088 (50 μg/mouse) was administrated via tail vein prior to instillation of mtDNA, and *in vitro* ODN2088 (25 μg) was added in the medium prior to mtDNA treatment. For CA074 Me (Millipore), *in vitro* MH-S cells were pre-treated with CA074 Me (100 μM, 40 μg) for 30 min before stimulation and then treated with CBNPs (100 µg/cm^2^) for 30 min with the presence of CA074 Me, and *in vivo* CA074 Me (40 μg/mouse) was administrated via tail vein 2 h prior to instillation of CBNPs. For SB 203580 (Sigma), *in vivo* SB 203580 (3.77 μg/mouse) was administrated via tail vein prior to instillation of CBNPs.

### LTB4 quantification

The quantification of LTB4 was performed using commercial EIA kits according to the manufacturer's instructions. LTB4 levels in cell culture supernatants and BALF were detected using LTB4 EIA Kit (Cayman Chemical).

### Statistical analysis

Statistical analysis was performed using GraphPad Prism 6. Significance was determined using a two-tailed unpaired Student's t-test. Data are presented as the mean ± SEM. P < 0.05 (*) was considered statistically significant (*P < 0.05; **P < 0.01; ***P < 0.001).

## Results

### Particle characterization

**Figure 1:**
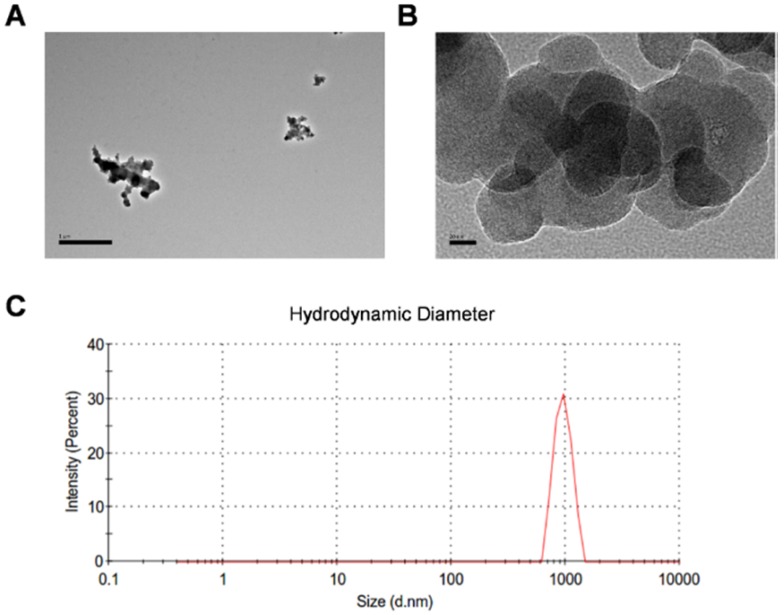
** Transmission electron microscopy images and dynamic light scattering analysis of CBNP instillation suspension. (A)** Different sized CBNP agglomerations ranging from nano- to µm-size. **(B)** High-resolution image of CBNPs. **(C)** The hydrodynamic number size-distribution analysis of CBNP instillation suspension.

CBNPs were obtained from Sigma as the manufacturer reported, with the primary particle being less than 100 nm, the specific surface area greater than 100 m^2^/g, and the carbon content higher than 99 wt%. The particle suspension was characterized by transmission electron microscopy (TEM) and dynamic light scattering analysis (DLS). The TEM image showed different degrees of agglomerated particles in the suspension (Figure 1A). The size-distribution plot showed a highly dominant size-mode with a peak-size at 955 nm and average size of 872.2 nm (Figure 1B). After sonication and vortex, CBNP dispersion had a moderate polydispersity index (PDI = 0.235). CBNPs had the low peak zeta-potential of -7.81 mV in instillation medium (conductivity 16.3 mS/cm) detected by DLS. Endotoxin level was less than 0.25 EU/mL (Figure S1).

### CBNPs induce acute pulmonary inflammation

**Figure 2:**
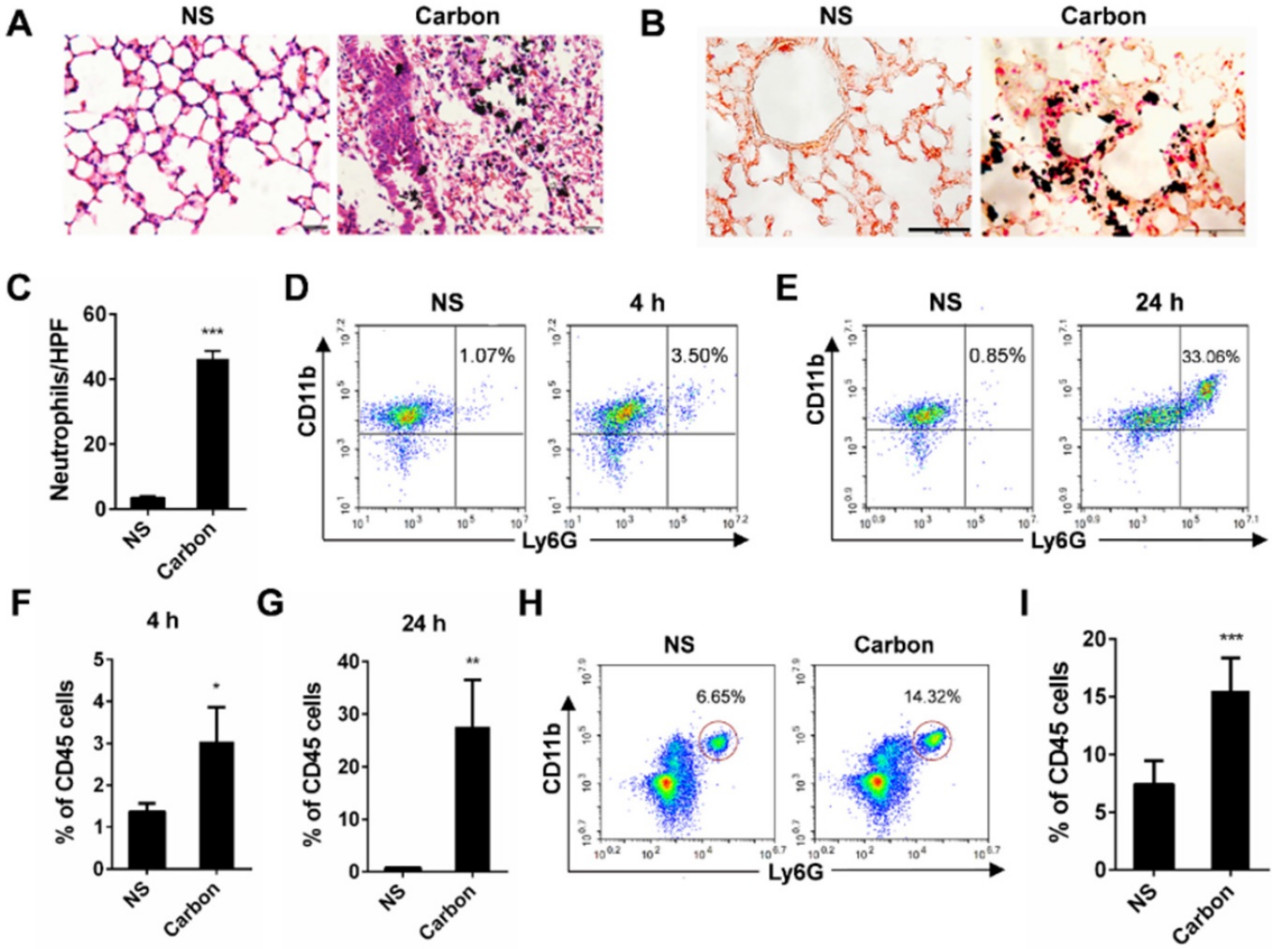
** CBNP instillation induces pulmonary neutrophilic inflammation.** C57BL/6 mice were instilled with saline containing 0.2 mg of CBNPs. After 24 h exposure, hematoxylin-eosin (H&E) staining **(A)** and specific esterase staining of neutrophils **(B)** in representative mouse lung sections are presented. (A) Scale bar = 20 µm; (B) Scale bar = 50 µm. **(C)** Esterase-positive neutrophils were counted in five high power fields (HPFs). Influxes of neutrophils in BALF at 4 **h (D, F)** and 24 h** (E, G)** were detected with flow cytometry by staining CD45, CD11b and Ly-6G. **(H-I)** The percentages of CD11b+Ly-6G+ neutrophils in CD45+ lung cells are shown 24 h later. Data are mean ± SEM; n = 3-6 per group. *P < 0.05, **P < 0.01, ***P < 0.001 compared with the control group by Student's t-test.

In the present study, we found that 24 h after a single instillation of CBNPs, mice exhibited pulmonary inflammation compared with the control group, as shown by haematoxylin-eosin (HE) staining (Figure 2A). This pulmonary inflammation was characterized by infiltrates of neutrophil-like cells (Figure S2), and esterase staining-positive cells were co-localized with the CBNP deposits (Figure 2B). The number of esterase-positive cells in each high-power field (HPF) of mouse lung section appreciably increased after a single instillation of CBNPs (Figure 2C). Additionally, neutrophil recruitment was confirmed by flow cytometry by labelling with anti-CD45, anti-CD11b and anti-Ly-6G antibodies. Neutrophil accumulation in bronchoalveolar lavage fluid (BALF) appeared as early as 4 h after CBNP instillation (Figure 2D and 2F) and remained considerably elevated at 24 h (Figure 2E and 2G). The number of neutrophils in whole lung digest samples increased 24 h after CBNP exposure (Figure 2H-I). Next, we investigated the underlying mechanism of CBNP-induced acute pulmonary neutrophilic inflammation.

### BNPs induce acute necrosis of alveolar macrophages* in vitro*

**Figure 3:**
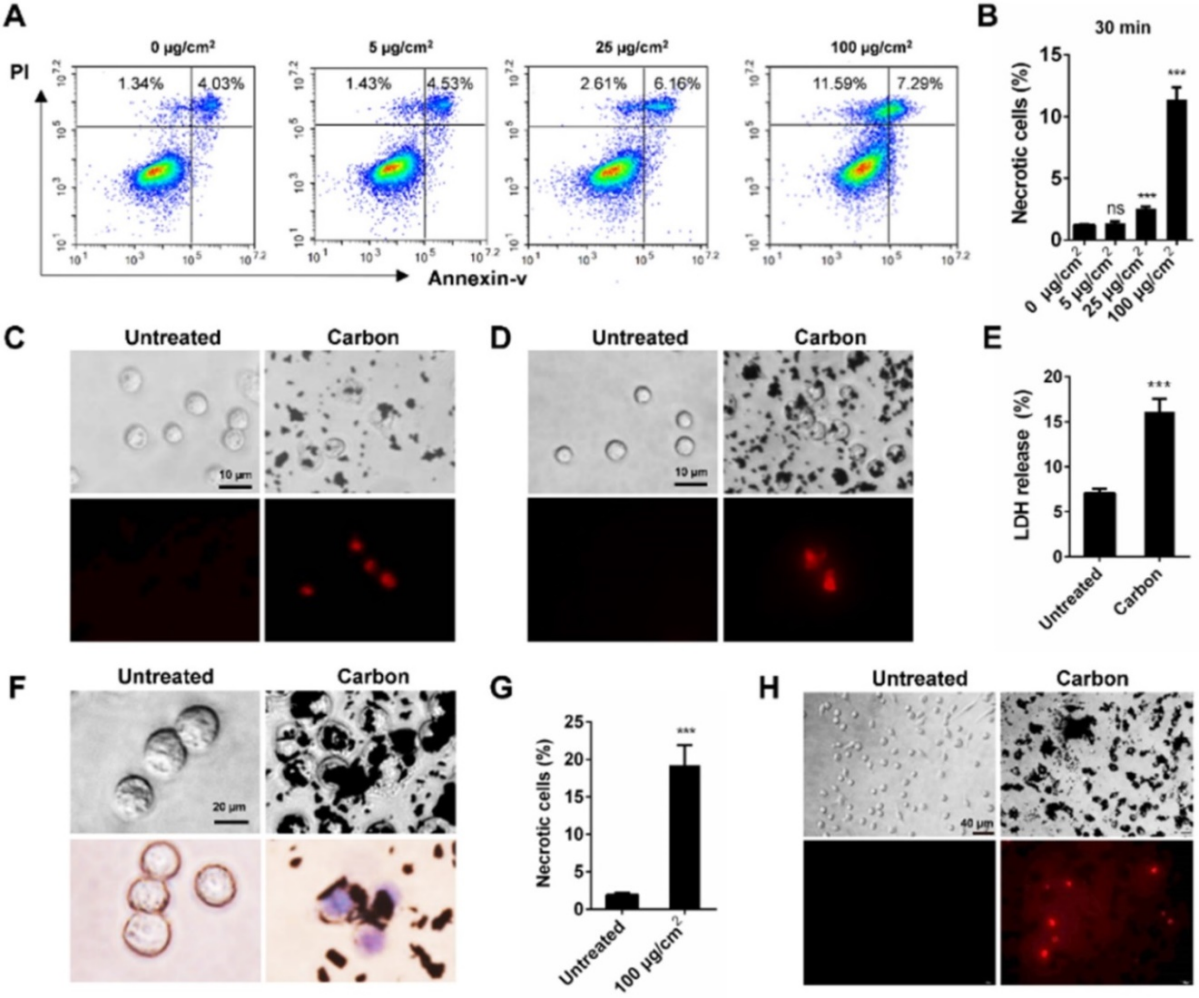
** CBNPs induce acute necrosis of alveolar macrophages *in vitro*. (A-B)** The detection of CBNP-induced necrotic cells *in vitro* by flow cytometry with Annexin V and PI staining. **(C)** Primary alveolar macrophages were treated with CBNPs (100 µg/cm^2^) for 30 min and then stained with PI. Scale bar = 10 µm. **(D)** Primary peritoneal macrophages were treated with CBNPs (100 µg/cm^2^) for 30 min and then stained with PI. Scale bar = 10 µm. **(E-F)** MH-S cells were treated with CBNPs at 100 µg/cm^2^ for 2 h. (E) Lactate dehydrogenase (LDH) release in culture supernatant was detected. (F) Morphological changes and trypan blue staining. Scale bar = 20 µm. **(G)** The percentages of necrotic cells after treatment with CBNPs at 100 µg/cm^2^ for 24 h detected by Annexin V/PI. **(H)** Peritoneal macrophages were treated with CBNPs at 100 µg/cm^2^ for 24 h and then stained with PI. Scale bar = 40 µm. Data are mean ± SEM; ns: no significance, ***P < 0.001 compared with control group by Student's t-test.

To elucidate the relationship between cell necrosis and inflammation induction, we tested whether CBNPs induced cell necrosis *in vitro* to exclude the contribution of inflammatory microenvironment to cell necrosis. Morphological change is the most direct evidence of cell death. The number of black particle-filled cells increased with increased dose and exposure time, and the excessive phagocytosis of particles led to cell swelling and lysis (Figure S3). Necrotic cells were detected by flow cytometry with PI and Annexin V staining. Cells in PI-positive and Annexin V-negative region are commonly recognized as necrotic cells. Cell necrosis is characterized by swollen cytoplasm and loss of plasma membrane integrity, followed by release of intracellular molecules 32. As early as 30 min, CBNPs induced cell necrosis in a dose-dependent manner (Figure 3A-B). CBNPs at a concentration of 25 µg/cm^2^ induced slight cell necrosis and a high dose of 100 µg/cm^2^ led to remarkably increased cell necrosis. Next, different assays were used to test the cytotoxicity induced by high-uptake of CBNPs (100 µg/cm^2^) in alveolar macrophages, peritoneal macrophages and MH-S cells at 30 min, 2h, and 24 h. Alveolar macrophages and peritoneal macrophages were exposed to CBNPs for 30 min, and as observed, the cells engulfed black particles and were positive for PI staining (Figure 3C and 3D). When the exposure period was extended to 2 h, cells were loaded with large amounts of black nanomaterials in the cytoplasm and exhibited apparent morphological changes, such as swollen cytoplasm and ruptured plasma membrane (Figure 3F). Particle-loaded macrophages suffered plasma membrane rupture and cell necrosis, confirmed by trypan blue staining (Figure 3F) and LDH release (Figure 3E). After exposure of 24 h, CBNPs caused a remarkable increase in the number of necrotic cells as detected by flow cytometry with PI and Annexin V staining (Figure 3G). After 24 h of treatment, the number of PI-positive peritoneal macrophages remarkably increased (Figure 3H).

### CBNP-induced cell necrosis is associated with lysosomal membrane permeabilization

**Figure 4:**
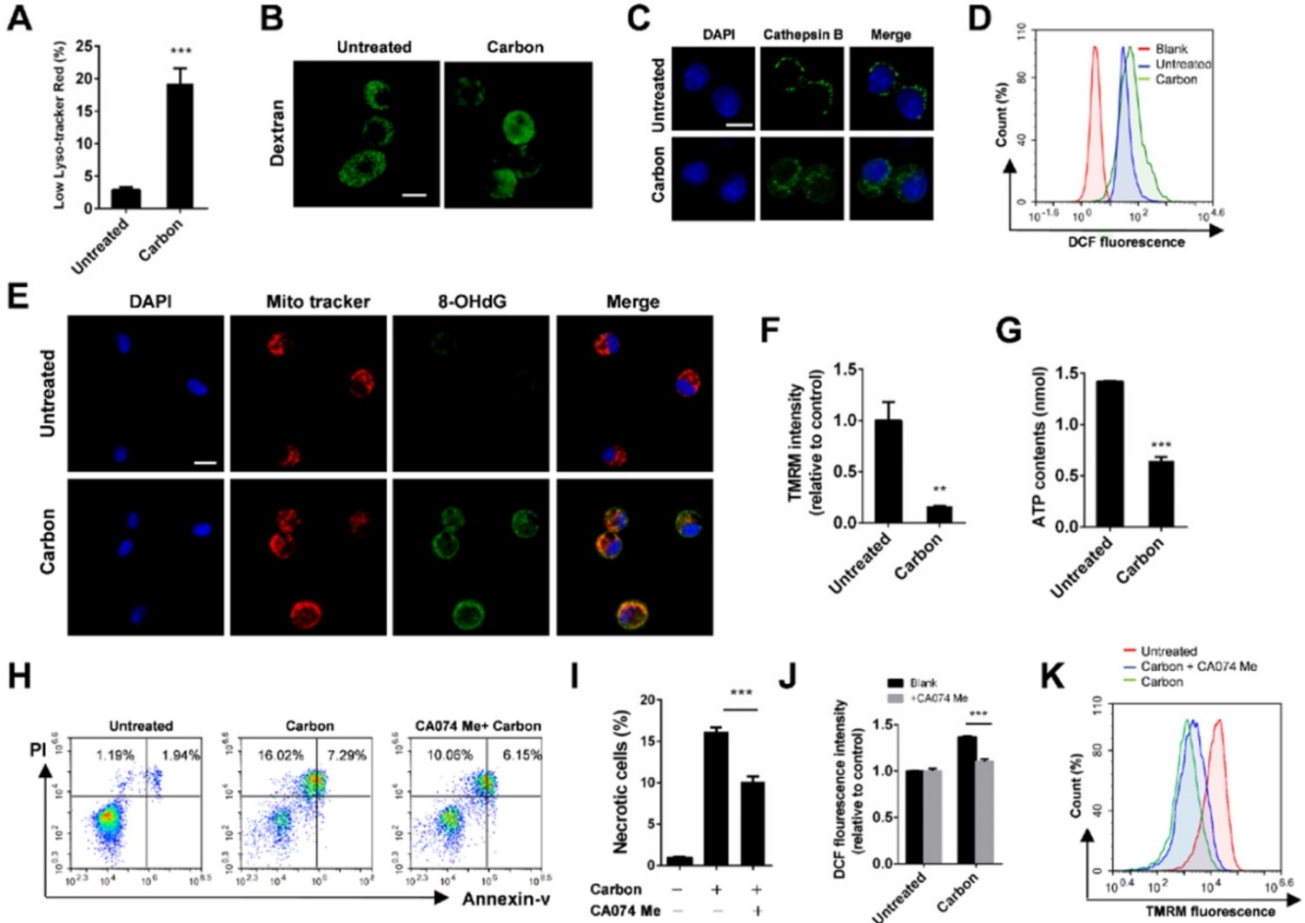
** CBNP-induced cell necrosis is associated with lysosomal membrane permeabilization. (A)** The fluorescence intensity of lysotracker-red in MH-S cells was detected by flow cytometry after treatment with CBNPs (100 µg/cm^2^) for 30 min. **(B)** MH-S cells were pre-loaded with 20-kDa FITC-conjugated dextran, and then treated with CBNPs (25 µg/cm^2^) for 2 h. **(C)** alveolar macrophages were treated with CBNPs (25 µg/cm^2^) for 2 h and then detected with mouse anti-mouse cathepsin B antibody. **(D)** The intracellular ROS levels were detected by flow cytometry after treatment with CBNPs (100 µg/cm^2^) for 30 min. **(E)** Mitochondrial DNA oxidative damage was detected by colocalization staining of Mito Tracker and 8-OHdG. **(F-G)** MH-S cells were incubated with CBNPs (100 µg/cm^2^) for 30 min. The mitochondrial membrane potential was measured with TMRM (F) and the cellular ATP levels (G) were detected with kits. **(H-K)** MH-S cells were pre-treated with CA074 Me (100 µM) for 30 min before stimulation and then treated with CBNPs (100 µg/cm^2^) for 30 min. The necrotic cells (H-I) were detected by flow cytometry. (J) Intracellular ROS levels were detected. (K) TMRM fluorescence intensity was detected. Scale bar = 10 µm. Data are mean ± SEM; n = 3 per group, **P < 0.01, ***P < 0.001 compared with the control group by Student's t-test

Considering that lysosome damage is often associated with biopersistent nanomaterial-induced cytotoxicity, we monitored lysosomal alterations of macrophages exposed to CBNPs. The lysosomes were labelled with lysotracker-red, a selective fluorescent probe that tracks acidic organelles in cells and generates punctate red fluorescence, and the decrease of fluorescence intensity indicates lysosomal damage. The percentages of cells with low lyso-tracker fluorescence intensity increased in a time- and dose-dependent manner after treatment with CBNPs (Figure S4). CBNPs at 25 µg/cm^2^ induced slight lysosomal damage after 30 min of treatment, which increased at 2 h. The high dose of 100 µg/cm^2^ led to remarkable lysosomal damage compared with the controls after 30 min of exposure (Figure 4A). Fluorescein isothiocyanate (FITC)-dextran (20 kDa) has been often used to detect lysosomal membrane permeabilization (LMP) 37. In the control cells, FITC-dextran was located in the lysosomes and presented bright green spots, while decreased amounts of fluorescent diffused throughout the cell interiors of the macrophages exposed to CBNPs (Figure 4B). Cathepsin B, a crucial lysosomal protease, was released into the cytoplasm, following the loss of the lysosomal membrane integrity in the treated cells. In contrast, the untreated macrophages showed dispersed fine granules (Figure 4C). The increased levels of intracellular reactive oxygen species (ROS) were detected by H2DCFDA oxidation assay after treatment with CBNPs (Figure 4D). Double labelling of Mito Tracker and DNA oxidation marker 8-OHdG in alveolar macrophages showed that the number of 8-OHdG-positive mitochondria increased after 2 h of exposure to CBNPs (Figure 4E). Healthy mitochondrial membranes maintain a difference in electrical potential between the interior and exterior of the organelle, named as mitochondrial membrane potential (MMP), a key indicator of mitochondrial function. The reagent Tetramethylrhodamine, Methyl Ester (TMRM) is often used to monitor changes in MMP and the changes in TMRM signal are detectable with flow cytometry. Mitochondrial matrix is at negative potential, and TMRM, as a cationic cell-permeant dye, accumulates in active mitochondria of healthy cells with intact membrane potentials. In this case, the fluorescence signal is bright. Upon loss of MMP, TMRM accumulation ceases and the signal dims or disappears 33,38. Mitochondrial membrane potential (ΔΨm) in MH-S cells was detected with TMRM by flow cytometry and the intracellular ATP levels were detected with colorimetric assays. Results showed that CBNPs caused reduced ΔΨm levels (Figure 4F) and ATP contents (Figure 4G). Pre-treatment with a specific cathepsin B inhibitor, CA074 Me (100 µM), reduced CBNP-induced cell necrosis (Figure 4H-I) and ROS generation (Figure 4J), and restored the loss of mitochondrial membrane potential (Figure 4K), indicating that Cathepsin B release worked upstream from these toxic endpoints.

### CBNPs induce acute necrosis of alveolar macrophages *in vivo*

**Figure 5:**
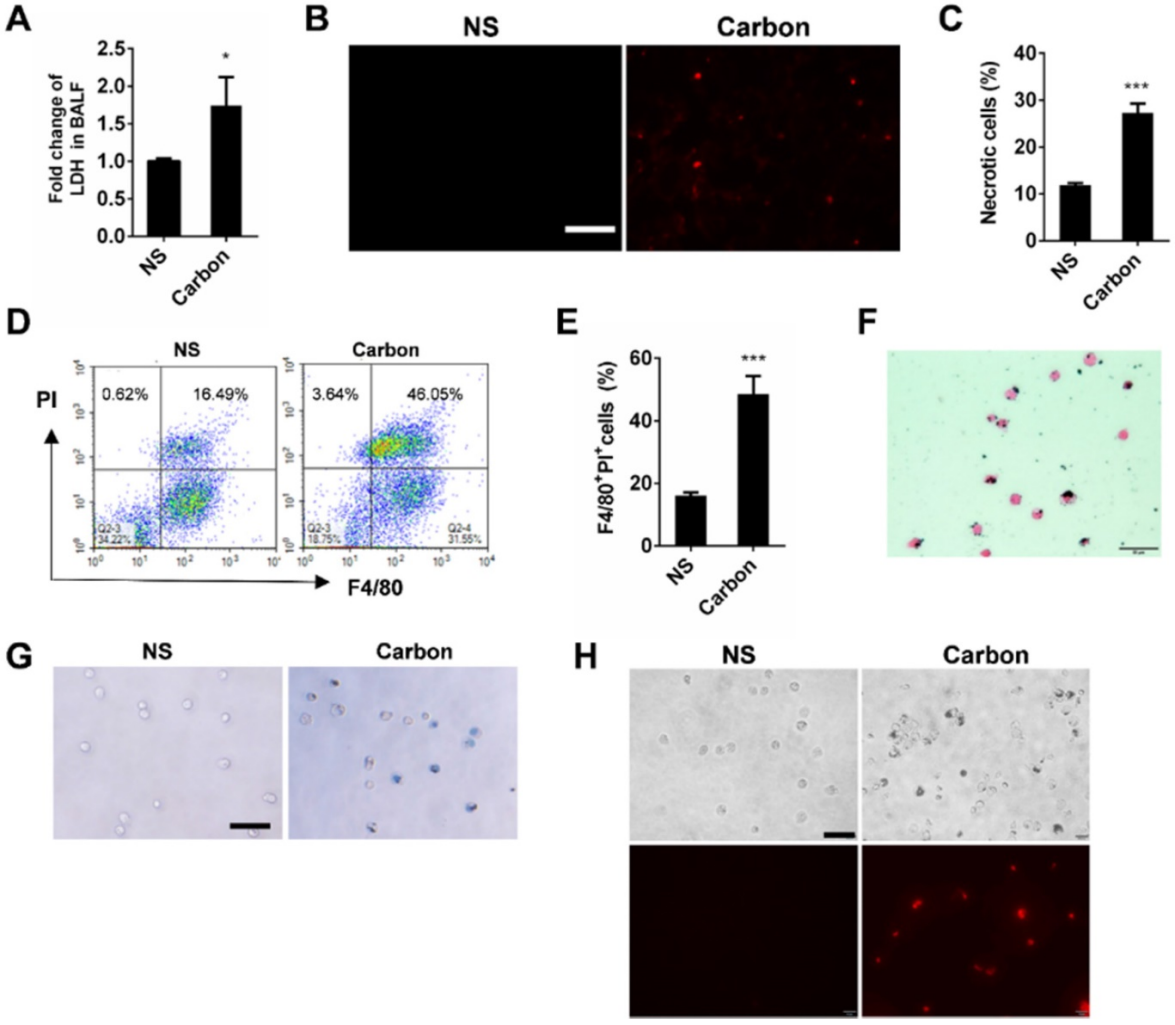
** CBNPs cause acute necrosis of alveolar macrophages* in vivo*.** C57BL/6 mice were exposed to a single instillation of saline containing 0.2 mg of CBNPs. **(A)** The detection of LDH release in BALF 30 min after instillation. **(B)** The representative images of PI-positive necrotic cells in mouse lungs. Scale bar = 50 µm. **(C)** The detection of the necrotic cells induced by CBNPs in BALF by flow cytometry with Annexin V and PI staining. Percentages of necrotic cells in PI-positive and Annexin V-negative region are shown. **(D-E)** Four hours after instillation, the detection of necrotic macrophages in BALF induced by CBNPs and the representative experiments by flow cytometry with PI and F4/80 staining are presented. **(F-H)** After 24 h of instillation, alveolar macrophages were obtained from the BALF of the exposed mice and the cells were stained by H&E (F), trypan blue (G) and PI (H). Scale bar = 50 µm. Data are mean ± SEM; n = 3 or 4 per group, *P < 0.05, ***P < 0.001 compared with the control group by Student's t-test.

Since we found that CBNPs induced acute necrosis of alveolar macrophages *in vitro*, we focused on how instilled CBNPs affect the lung tissue shortly after administration, and investigated whether CBNPs induce cell death *in vivo*, hoping to link CBNP-induced cell necrosis and acute pulmonary neutrophilic inflammation. The LDH levels in the BALF of exposed mice increased as early as 30 min after exposure (Figure 5A), indicating that early lung injury involving cell death was caused by the treatment of CBNPs. PI staining *in vivo* was performed for the detection of necrotic cells within lung tissues after 4 h of instillation, and PI-positive cells were observed in mouse lungs (Figure 5B). The cells in PI-positive and Annexin V-negative region are commonly recognized as necrotic cells. The number of necrotic cells increased in BALF 4 h after instillation, detected by flow cytometry with PI and Annexin V staining (Figure 5C). The cell necrosis of macrophages in BALF was detected by double staining with PI and F4/80. CBNP instillation led to a remarkable increase in the number of necrotic macrophages (Figure 5D-E). After 24 h of CBNP instillation, alveolar macrophages were harvested from BALF of the exposed mice. The cells were black particle-loaded (Figure 5F and Figure S5), and suffered plasm membrane rupture and cell necrosis, indicated by trypan blue (Figure 5G) and PI staining (Figure 5H).

### CBNPs induce pulmonary inflammation through the mitochondria release from necrotic cells

**Figure 6:**
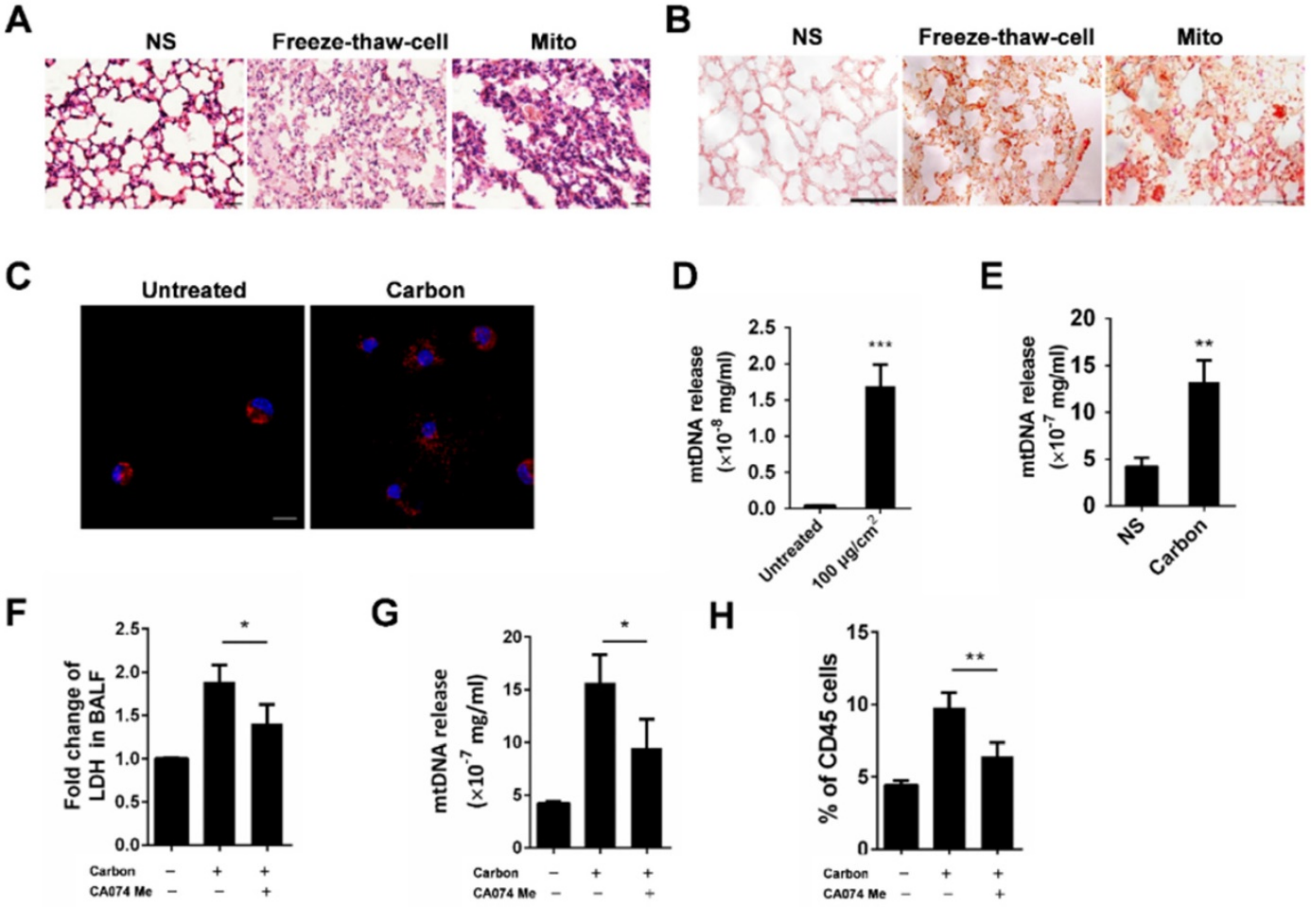
** Pulmonary inflammation triggered by necrotic cells and the released mitochondria. (A-B)** The alveolar macrophages were treated with freeze-thaw process for necrosis. Mitochondria were extracted from the lungs of C57BL/6 mice. The treated cells and extracted mitochondria were instilled into C57BL/6 mice (cells: 10^6^/mouse, mitochondria: 200 µg/mouse). HE staining (A) and specific esterase staining of neutrophils (B) in representative mouse lung sections 24 h after exposure are presented. (A) Scale bar = 20 µm; (B) Scale bar = 50 µm. **(C)** Mito Tracker-stained alveolar macrophages were treated with CBNPs (25 µg/cm^2^) for 2 h and then subjected to the observation of mitochondria leakage (mitochondria: red; DAPI: blue). Scale bar = 10 µm. **(D)** MH-S cells were treated with CBNPs (100 µg/cm^2^) for 4 h and the released mtDNA in the culture supernatant was determined by qPCR. **(E)** C57BL/6 mice were instilled with saline containing 0.2 mg of CBNPs. Four hours later, the supernatant of BALF was collected for mtDNA determination by qPCR. **(F-H)** The mice were pretreated with CA074 Me prior instillation of CBNPs. Four hours later, the LDH release (F) and mtDNA release (G) in BALF was detected, and 24 h later, neutrophil percentages in lungs were detected (H). Data are mean ± SEM; n = 3 or 4 per group. *P < 0.05, **P < 0.01, ***P < 0.001 compared with the control group by Student's t-test.

Since acute cell necrosis was induced by CBNPs, we postulated that endogenous signal molecules are released from necrotic cells and cause subsequent lung injury. We investigated whether necrotic alveolar macrophages induce lung inflammation in mice. Mice were intratracheally instilled with necrotic alveolar macrophages prepared by a freeze-thaw process, and severe pulmonary inflammation was observed by HE and esterase staining 24 h after instillation (Figure 6A-B). Mitochondria-derived DAMPs have been recently reported as key DAMP member, exhibiting immune stimulatory capacities 29. The mice were instilled with extracted mitochondria and pulmonary inflammation was induced (Figure 6A and Figure S6), indicating the important role of released mitochondria in CBNP-induced pulmonary inflammation. The number of esterase-positive cells was markedly greater in the mitochondria-instilled lungs than the controls (Figure 6B). The leakage of mitochondria from CBNP-treated macrophages was detected by labelling cells with Mito Tracker. After incubation with CBNPs, necrotic cells released mitochondria (Figure 6C), and the release of mtDNA in culture supernatant was confirmed by real-time quantitative PCR (qPCR) (Figure 6D). Additionally, increased amounts of mtDNA were detected in the BALF of treated mice (Figure 6E). We postulated that CBNP-induced lung inflammation was associated with cell damage. *In vitro*, it has been tested that CA074 Me blocked CBNP-induced cell necrosis (Figure 4H-I). Next, we investigated whether CA074 Me worked *in vivo*. The pretreatment with CA074 Me reduced LDH and mtDNA release in BALF caused by CBNPs (Figure 6F and 6G). Furthermore, CA074 Me inhibited the infiltration of neutrophils in lungs (Figure 6H).

### Neutrophils are activated by mtDNA and formyl-peptides

**Figure 7:**
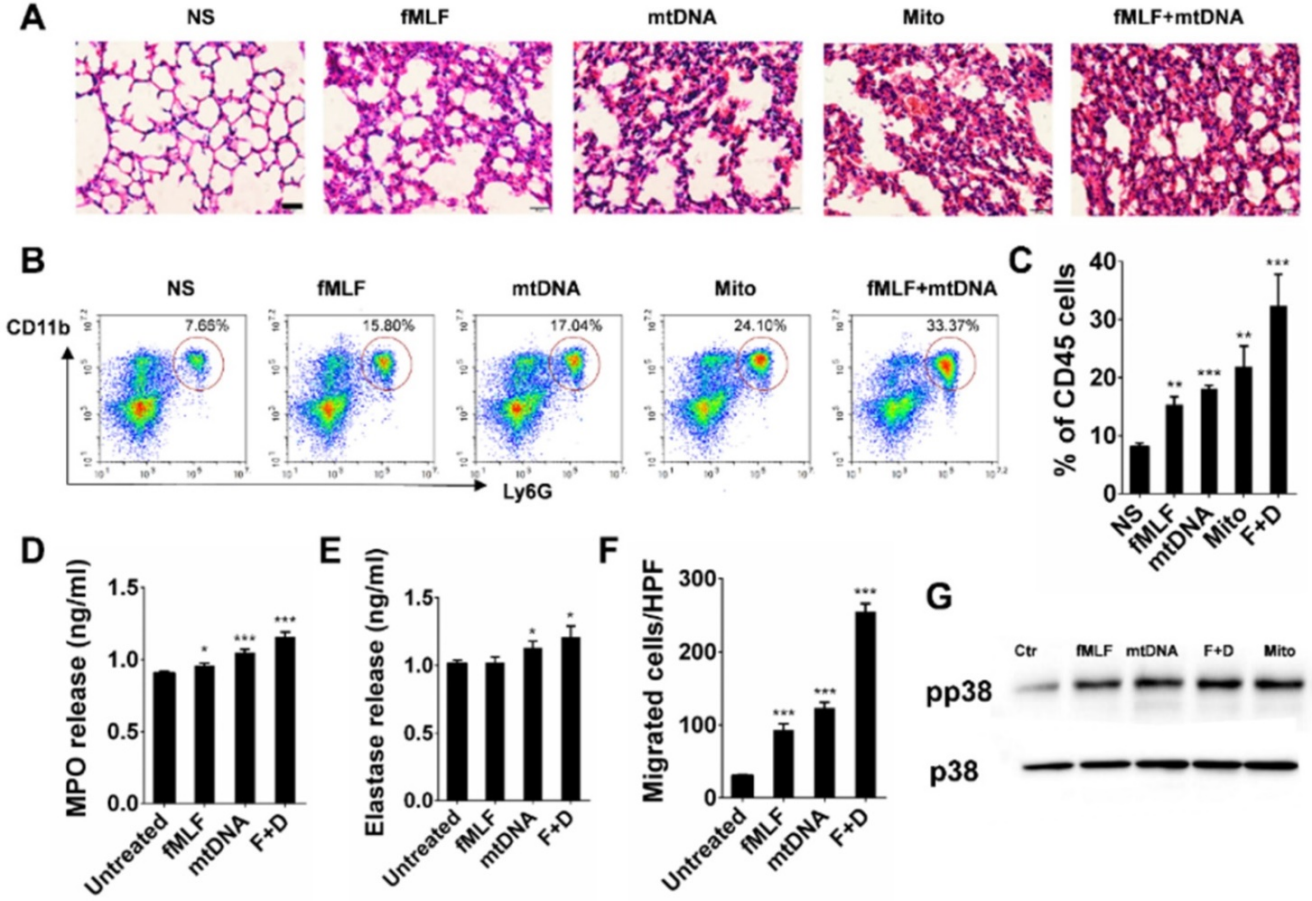
** Neutrophil activation triggered by mtDNA and fMLF. (A-C)** C57BL/6 mice were instilled with normal saline, fMLF (1 µM, 200 µl), mtDNA (2.5 µg) and mitochondria (200 µg), and were sacrificed 24 h after instillation. n = 3 per group. (A) The representative lung sections of HE staining are shown. Scale bar = 20 µm. (B) Influx of neutrophils in whole lungs was detected with flow cytometry by labelling CD45, CD11b and Ly-6G. (C) The percentages of CD11b+Ly-6G+ neutrophils in CD45+ cells of the lungs are shown. **(D-E, G)** Isolated murine neutrophils (5 × 10^6^) were incubated with fMLF (1 µM), mtDNA (2.5 µg/ml) and mitochondria (200 µg/ml) for 2 h *in vitro*. n = 3 per group. The release of MPO (D) and elastase (E) in the supernatant of neutrophils was determined. **(F)** Neutrophil chemotaxis assays were performed with fMLF (1 µM) and mtDNA (2.5 µg/ml) for 4 h and the number of migrated cells was counted in five HPFs. (G) Western blotting was performed to confirm the activation of p38 MAPK in neutrophils induced by fMLF and mtDNA. Data are mean ± SEM; *P < 0.05, **P < 0.01, ***P < 0.001 compared with the control group by Student's t-test.

The mitochondria released from necrotic cells led to the infiltration of neutrophil-like cells. Furthermore, we investigated the possible activation pathways of cultured neutrophils mediated by mitochondrial components. Severe pulmonary inflammation was observed by HE staining after the instillation of isolated mitochondria, mtDNA and synthetic peptide N-formyl-Met-Leu-Phe (fMLF) (Figure 7A). In parallel, neutrophil recruitments in the corresponding lung tissues were detected by flow cytometry (Figure 7B-C). The MPO and elastase levels in the culture supernatant of isolated mouse neutrophils increased after 2 h of incubation with mtDNA and fMLF, whereas mtDNA caused more release of MPO and elastase (Figure 7D-E). The mtDNA and fMLF activated the p38 mitogen-activated protein kinase (MAPK) pathway and caused the phosphorylation of neutrophil p38 (Figure 7G). In addition, we evaluated the effects of mtDNA and fMLF on neutrophil migration. As a result, neutrophil migration increased after mtDNA and fMLF treatment (Figure 7F). Thus, mtDNA mediates neutrophil activation involved in p38 phosphorylation, MPO and elastase release, and fMLF plays a similar role to that of mtDNA in neutrophil activation.

### TLR9-p38 pathway is involved in mtDNA- and CBNP-induced pulmonary inflammation

**Figure 8:**
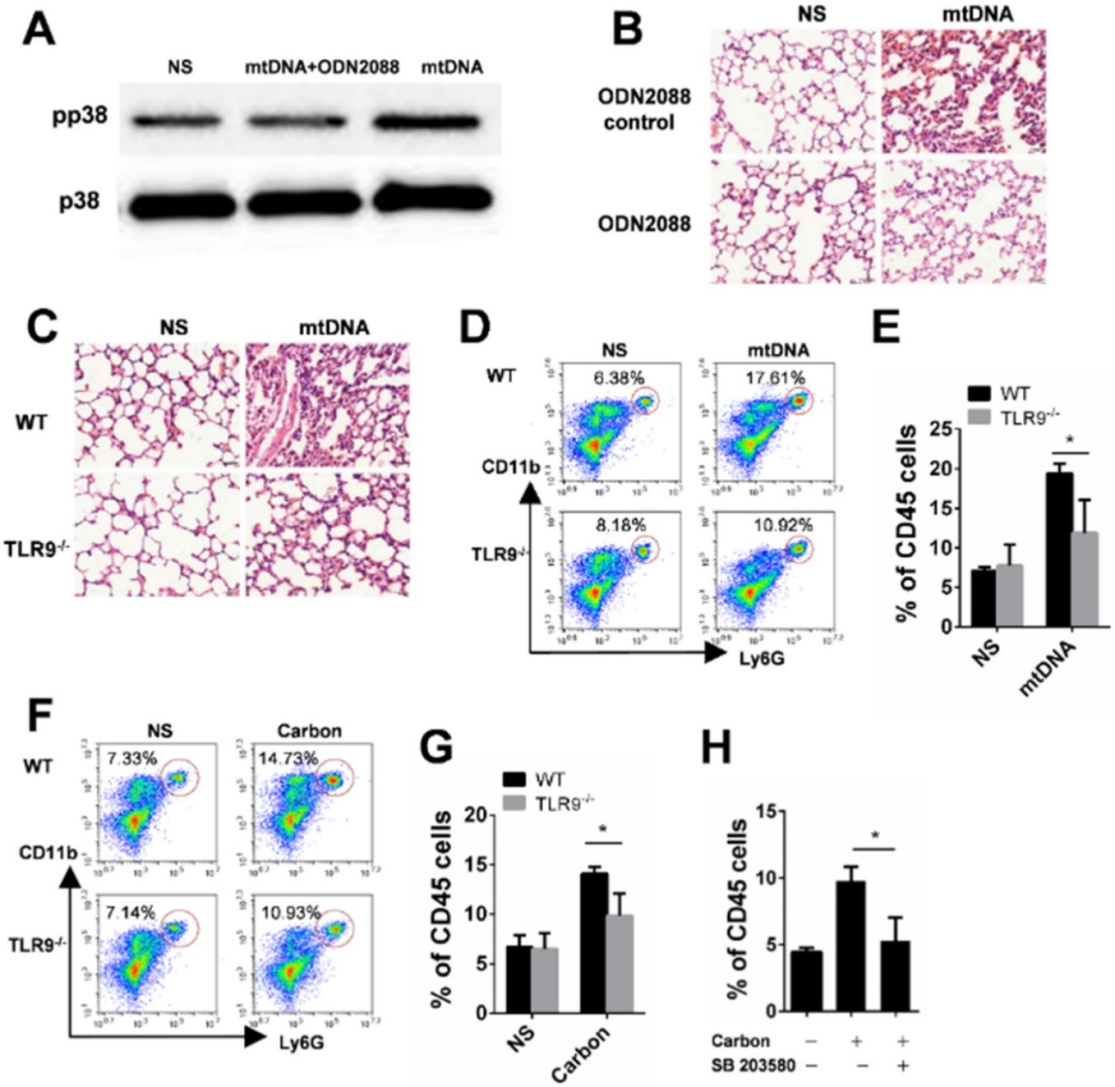
** TLR9-p38 pathway involvement in mtDNA- and CBNP-induced pulmonary inflammation. (A)** ODN2088 (25 µg) pretreatment inhibited p38 phosphorylation of neutrophils induced by mtDNA *in vitro*. **(B)** Mitochondrial DNA (2.5 µg) with the pretreatment of ODN2088 or ODN Control (50 µg/mouse) was instilled into wide-type C57BL/6 mice. H&E staining of mouse lung sections 24 h after instillation is shown. Scale bar = 20 µm. **(C)** The representative lung sections for the H&E staining of wild-type C57BL/6 mice and Tlr9^-/-^ mice are shown. Scale bar = 20 µm. **(D-E)** The influxes of neutrophils in the whole lungs of wild-type C57BL/6 mice and Tlr9^-/-^ mice were detected 24 h after mtDNA instillation. **(F-G)** The influxes of neutrophils in the whole lungs of wild-type C57BL/6 mice and Tlr9^-/-^ mice were detected 24 h after CBNPs instillation. **(H)** Prior instillation of CBNPs, mice were pretreated with SB 203580, 24 h later the influxes of neutrophils in the whole lungs were detected. Data are mean ± SEM; n = 3-4 per group. *P < 0.05 by Student's t-test.

In the present study, we found that CBNP-induced pulmonary inflammation was related with mtDNA release, and the immunostimulatory capacity of mtDNA was attributed to an abundance of unmethylated CpG motifs that share similarities with bacterial DNA 39,40. The TLR9-MyD88 pathway reportedly plays an important role in mtDNA-induced lung inflammation 32. ODN2088, a TLR9 antagonist, inhibited p38 phosphorylation in neutrophils induced by mtDNA *in vitro* (Figure 8A), and the inflammatory response induced by mtDNA was alleviated after pretreatment with ODN2088 (Figure 8B). We further used gene knockout mice to identify the pathway of mtDNA- and CBNP-induced neutrophil infiltration. Mito DNA were intratracheally instilled into wild-type (WT) and TLR9^-/-^ mice, and mtDNA-induced inflammatory response (Figure 8C) and neutrophil infiltration (Figure 8D-E) in the lungs of TLR9^-/-^ mice were attenuated. CBNPs were intratracheally instilled into WT and TLR9^-/-^ mice, and CBNP-induced neutrophil infiltration (Figure 8F-G) in the lungs of TLR9^-/-^ mice were reduced. We further demonstrated that pretreatment with p38 inhibitor SB 203580 could reduce neutrophil aggregation induced by CBNPs (Figure 8H). The results suggested that the TLR9-p38 pathway might play an essential role in the induction of lung inflammation by CBNPs or mtDNA.

### The released mtDNA induces LTB4 production from macrophages

**Figure 9:**
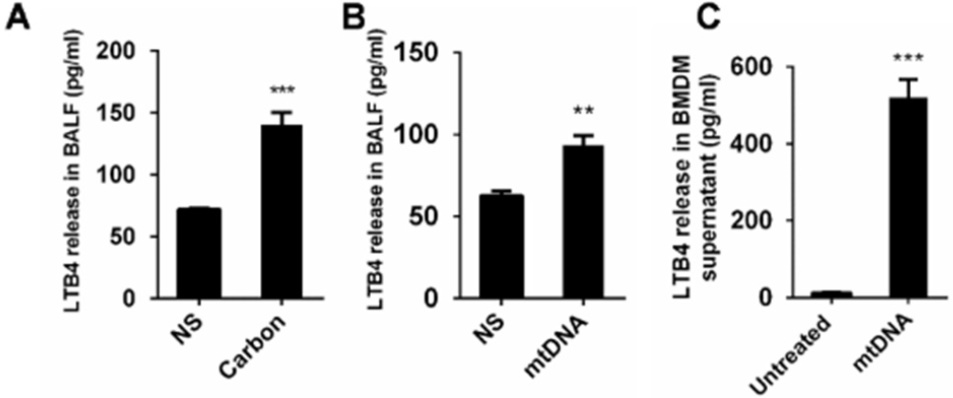
** The released mtDNA induces LTB4 production from macrophages. (A-B)** C57BL/6 mice were instilled with saline containing 0.2 mg of CBNPs or mtDNA (2.5 µg). After 6 h of treatment, the supernatant of the BALF was collected for LTB4 determination (n = 3 per group). **(C)** Bone marrow-derived macrophages (BMDMs) were primed with LPS (10 ng/ml) for 3 h and then treated with mtDNA (2.5 µg) for 6 h. The cell culture supernatants were collected for LTB4 determination. Data are mean ± SEM; **P < 0.01, ***P < 0.001 by Student's t-test.

The Lipid chemoattractant leukotriene B4 (LTB4) levels in the BALF of mice exposed to CBNPs were increased (Figure 9A). We demonstrated that high uptake of CBNPs induced acute cell necrosis of macrophages, and the released mtDNA from necrotic cells activated neutrophils and triggered pulmonary inflammation. Thus, we postulated that LTB4 production is likely ascribed to CBNP-induced mtDNA release. As expected, the LTB4 levels in the BALF of mice exposed to mtDNA were increased (Figure 9B) and mtDNA significantly increased LTB4 production *in vitro* (Figure 9C).

## Discussion

**Figure 10:**
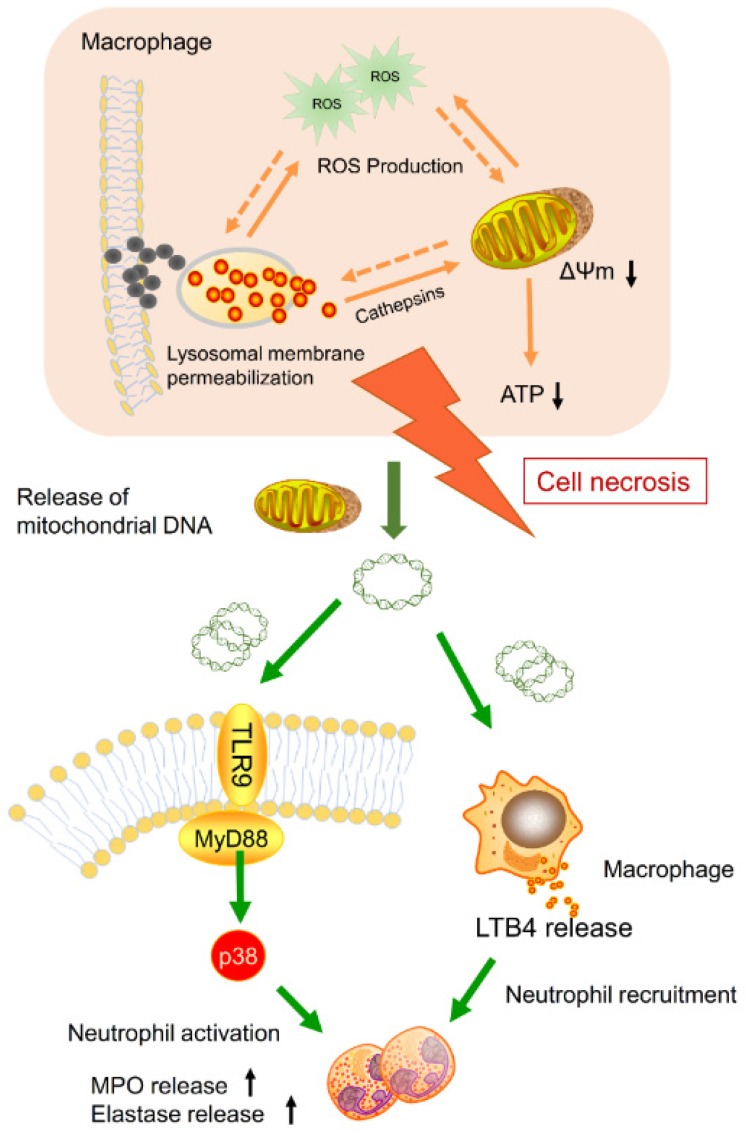
** Model of the induction of pulmonary neutrophilic inflammation caused by mtDNA release from CBNP-induced necrotic alveolar macrophages.** CBNPs induced acute cell necrosis of alveolar macrophages involving lysosomal dysfunction, ROS generation and energy depletion. Cell necrosis caused the subsequent release of mitochondria and mitochondrial contents, such as mtDNA which emerge as important members of damage-associated molecular patterns (DAMPs). Mitochondrial DNA activated the p38 MAPK and triggered the LTB4 production, thus recruiting neutrophils and inducing pulmonary inflammatory injury.

Nanoparticle inhalation has been extensively shown to contribute to respiratory morbidity and mortality 20,21,41. As a primary component in air pollution, CBNPs have been increasingly investigated on their toxicity. A better understanding of the mechanism of CBNP-induced toxicity may allow for the appropriate regulation of potential health hazards. Previous studies have implicated the important role of direct and indirect ROS generation in carbon nanomaterial-related inflammation by particle-cell interactions 42. In this study, we revealed a novel mechanism concerning with CBNP-induced cell necrosis of alveolar macrophages and acute lung inflammation. We found that a single instillation of CBNPs induced neutrophil influx into lung as early as 4 h post-exposure that began with the rapid appearance of necrotic macrophages. Macrophages could take up inhaled nanoparticles and undergo acute cell necrosis, which was associated with lysosome rupture, ROS generation and mitochondrial dysfunction. The mtDNA released from the necrotic macrophages mediated pulmonary neutrophilic inflammation. Additionally, macrophages responded to mtDNA via the release of inflammatory mediator LTB4 (Figure 10).

A number of studies explored the cytotoxicity of CBNPs with inconsistent outcomes. It was reported that CBNPs induced LDH release in a dose (3-30 µg/cm^2^) and time (4-24 h) course, and the way of cell death was identified as pyroptosis, an inflammasome-dependent form of cell death, defined by inflammasome activation, cleavage of caspase 1 and downstream IL-1β release 23. Another study reported that CBNPs (10-40 µg/cm^2^) caused significant loss of ΔΨm and ROS generation as early as 30 min 43. In contrast, one study showed that carbon black did not induce LDH release 13. In this study, we identified the cytotoxicity of CBNPs (5-100 µg/cm^2^) in a time- and dose-dependent manner. After treatment for 30 min, 5 µg/cm^2^ of CBNPs did not induced cytotoxicity, 25 µg/cm^2^ of CBNPs induced slight cell necrosis, and CBNPs at 100 µg/cm^2^ induced significantly increased cell necrosis. As the exposure time prolonged, excessive particles could be fully engulfed by cells, leading to cytoplasm swelling and lysis. As one previous paper reported 44, a total of 2.9×10^6^ alveolar macrophages were located in the lungs per mouse. The dose of 25 μg/cm^2^ *in vitro* might equal to the *in vivo* dose of 200 μg/mouse, as calculated from a total number of exposed cells in 24-well plates (3.5×10^5^/cm^2^). The dose level of 25 µg/cm^2^ is moderate and has been widely used in previous literatures. The exposure dose of 100 µg/cm^2^ is relatively large, but is also within the range used in previous studies, in which a high dose of 500 µg/ml was adopted for the evaluation of carbon nanoparticle-induced toxicity 45.

In the experiments focused on the toxicological mechanisms, the percentages of cells with low lyso-tracker fluorescence intensity increased after treatment with CBNPs in a time- and dose-dependent manner. Two exposure protocols were chosen (i.e., 25 µg/cm^2^ for 2 h and 100 µg/cm^2^ for 30 min) to verify the consistent mechanism of cytotoxicity induced by high and low doses of CBNPs. We observed the toxic effects on macrophages exposed to high dose for 30 min, and these effects extended to the lower dose group for 2 h of exposure.

After endocytosis by phagocytic or non-phagocytic mechanisms, nanomaterials often end up with lysosome internalization 37. Lysosomes are membrane-enclosed organelles with acidic pH and variety of hydrolytic enzymes (e.g., proteases, phosphatases, nucleases, and lipases), representing an extremely hostile environment that degrades all but some biopersistent nanomaterials, such as carbon black nanoparticles 46. Excessive CBNPs are accumulated in lysosomes, presumably leading to lysosomal swelling and rupture. The cysteine protease Cathepsin B and the aspartic protease Cathepsin D are two key hydrolytic enzymes 47, involved in lysosomal membrane permeabilization-related cell death 48. Mitochondria are both the major intracellular source and target of oxidative stress, and mitochondrial dysfunction further induced ROS generation. During LMP, lysosomal cathepsins were released into the cytosol, triggering mitochondrial dysfunction and promoting ROS generation 49-52. Intracellular ROS resulted in oxidative damage to DNA, proteins and lipids, and fed back to amplify the mitochondrial dysfunction and lysosomal membrane damage 53-56. Double labeling of Mito Tracker and 8-OHdG was used to affirm mtDNA oxidation as previous reported 57. Mitochondrial DNA is more sensitive to ROS-induced damage compared with nuclear DNA because of a lack of protective histones and diminished DNA repair capacity 58. Pretreatment with a specific Cathepsin B inhibitor, CA074 Me, decreased CBNP-induced necrosis and ROS generation, and CA074 Me restored CBNP-induced mitochondrial membrane potential loss, indicating that Cathepsin B worked upstream from these cellular endpoints. Therefore, we concluded that lysosomal rupture and Cathepsin B release played a key role in CBNP-induced cellular toxicity.

In this study, we adopted several methods to detect LMP, including evaluation of the cellular distribution of acidotropic dye (Lyso-tracker Red), lysosomotropic particles (FITC-dextran) and immunostaining of lysosomal protein (Cathepsin B). The release of lysosomal cathepsin proteases, serving as broadly specific hydrolytic enzymes, strongly impacts cellular physiology. They are mainly responsible for the degradation of endocytosed extracellular matrix components, autophagocytosed proteins and intracellular multi-protein structures such as organelles. The broad degradation targets of cathepsins could lead to mitochondrial dysfunction, osmotic imbalance and ultimate loss of cellular structural integrity and cell necrosis 59,60. In the study, we observed CBNP-induced cell membrane rupture and mtDNA release, which might be triggered by released cathepsins.

Our *in vivo* findings corroborated two earlier studies demonstrating CBNP-induced lung inflammation. Multiple doses (i.e., 0.018, 0.054 and 0.162 mg) were administrated by intratracheal instillation, and the neutrophil infiltration in BALF peaked 1 day postexposure, and the two higher doses of CBNPs induced the decrease of macrophages in BALF at 24 h. It was also demonstrated that CBNPs could cause genotoxicity both in lungs and a secondary tissue, the liver 20. In another study, 0.054 mg of CBNPs was used, and the results showed the infiltration of neutrophils and decreased number of macrophages in BALF 21. The relationship between neutrophil infiltration and macrophage necrosis has not been reported. In this study, we demonstrated that CBNPs induced cell necrosis through lysosomal rupture and subsequent lung inflammation mediated by released mtDNA from necrotic cells. Although the exposure dose is relatively large, it is also within the range used in the previous study of CBNPs toxicology 61. It has been reported that respirable carbon black in the work environment might be up to 2.5 mg/m^3^ 62. The high dose (200 μg) may represent a substantial dose for respirable carbon black in workplace. The dose of 200 μg/mouse in our study equals the approximate pulmonary deposition of the workers after 5 day-exposure, as calculated from a mouse at the occupational exposure of carbon black of 2.5 mg/m^3^ with a volume of inhaled air per hour of 1.8 L/h 63 based on the assumption that 33.8% of the inhaled mass deposited in the pulmonary region 21. Furthermore, in order to understand the toxic effects of CBNPs in the level of daily exposure dose, we established *in vivo* experiments with a low dose of 0.01 mg. It equals to the pulmonary deposition for 5 days of inhalation exposure in severe air pollution (PM_2.5_ ≥ 250 μg/m^3^) based on a deposition rate of 20% for PM_2.5_ 64. CBNPs at the low dose of 0.01 mg induced LDH release in BALF at 4 h, and mtDNA release in BALF at 12 h, and slight neutrophil infiltration in lungs at 24 h (Figure S7). Future works are needed to establish repeated chronic daily inhalation to more closely mimic human exposures.

Alveolar macrophages, as the primary cell type clearing inhaled particulates, are representative of the lung target of CBNPs. Intratracheal instillation of CBNPs induced cell damage, registered in BALF as early as 30 min post-exposure. The number of necrotic F4/80 (+) cells significantly increased in the BALF of treated animals and neutrophil influxes were observed as early as 4 h post-exposure. It has been well documented that the release of DAMPs following cell necrosis mediated inflammatory responses. In the present study, the release of mitochondria was detected in CBNP-treated cultured cells with Mito Tracker staining. Increased concentrations of mtDNA in cell supernatant and BALF after CBNP treatment were determined by qRT-PCR. The mitochondria and mitochondrial contents released from necrotic alveolar macrophages mediated CBNP-induced acute pulmonary inflammation. The administration of freeze-thaw cells, isolated mitochondria, mtDNA and fMLF also induced severe pulmonary inflammation. MPO, as a highly oxidative enzyme, is a key mediator of the damaging innate immune response 65. The mtDNA triggered neutrophil activation through increased release of MPO and elastase. Additionally, the p38 MAPK pathway of neutrophils was activated by the mtDNA. Moreover, the mtDNA-mediated pulmonary inflammation was related to the TLR9 pathway, confirmed by using TLR9^-/-^ mice and TLR9 antagonist ODN2088. The TLR9^-/-^ mice and mice pretreated with ODN2088 exhibited reduced inflammation. Based on our results, mitochondria were released from necrotic cells due to CBNPs, and mtDNA and formyl-peptides triggered neutrophilic inflammation through TLR9 pathway. Other DAMPs such as high mobility group box 1 (HMGB1) and uric acid may also be released following cell necrosis 66, and whether these DAMPs play a role in CBNP-induced inflammation is an intriguing question to be further explored.

Chemokines orchestrate the process of inflammatory cell recruitment to sites of tissue injury and LTB4 is one of the early mediators of inflammation 67,68. In inflamed lungs, neutrophil-active cytokines may be produced from lung resident cells or recruited leukocytes 36. LTB4 is one of the early mediators of inflammation, and crystalline silica reportedly induced LTB4 production by macrophages, which contributed to neutrophil recruitment 36. In this work, we found that mtDNA induced LTB4 production by macrophages that could possibly contribute to neutrophil recruitment.

Nevertheless, several concerns regarding our study might be addressed. Poor dispersion of CBNPs might pose a challenge to the consistency of the results in the toxicity evaluation both *in vivo* and *in vitro*. CBNPs are water insoluble and which tended to aggregate in aqueous solution, resulting in the larger sizes and smaller surface areas. In the previous studies, surfactants and Tween 80 were used to prepare well-dispersed carbon nanomaterials 69,70; however, these additional surfactants could cause an increase in the intracellular ROS in A549 cells reportedly 71. Therefore, in the present study, we suspended high-purity CBNPs in endotoxin-free normal saline with 30 min of sonication for de-agglomeration. We focused on the biological effects caused by the material and investigated how alveolar macrophages and the lungs respond to the inhaled CBNPs.

## Conclusion

Overall, this work demonstrates that CBNPs induce cell necrosis through lysosomal membrane permeabilization and alveolar macrophages play critical roles in CBNP-induced lung inflammation. Alveolar macrophages were identified to play a key role in the induction of lung inflammatory responses to CBNP exposure, in which particles could induce necrosis of alveolar macrophages and induce the release of mtDNA and inflammatory cytokines. The released DAMPs from necrotic alveolar macrophages mediated neutrophilic inflammation through the TLR9 pathway and LTB4 production. These findings might be of some importance in illustrating how the increased air pollutant particles affect human health and also throw some light on the possible prevention and treatment target for particle-associated lung diseases. The role of mtDNA in particulate-related pulmonary inflammation in humans needs to be further verified for developing potential strategies to prevent unwanted inflammatory response.

## Supplementary Material

Supplementary figures.

## Abbreviations

CBNPs: carbon black nanoparticles
NP: nanoparticle
PM: particulate matter
DAMPs: damage-associated molecular patterns
mtDNA: mitochondrial DNA
BALF: bronchoalveolar lavage fluid
LMP: lysosomal membrane permeabilization
ROS: reactive oxygen species
fMLF: N-formyl-Met-Leu-Phe
MPO: myeloperoxidase
LTB4: leukotriene B4
LDH: lactate dehydrogenase
TMRM: tetramethylrhodamine methyl ester
