# Supplementary material for: Carbon black nanoparticles induce cell necrosis through lysosomal membrane permeabilization and cause subsequent inflammatory response
Source: Theranostics. 2020 Mar 15;10(10):4589–605. doi: 10.7150/thno.34065 (PMC7150486; doi:10.7150/thno.34065)
Supplement: Supplementary file 1 — Supplementary figures. [file thnov10p4589s1.pdf]

Carbon black nanoparticles induce cell necrosis through lysosomal membrane permeabilization and cause subsequent inflammatory response

Xia Yuan, Wen Nie, Zhiyao He, Jingyun Yang, Bin Shao, Xuelei Ma, Xiangxian Zhang, Zhenfei Bi, Lu Sun, Xiao Liang, Yan Tie, Yu Liu, Fei Mo, Dan Xie, Yuquan Wei, Xiawei Wei*

Laboratory of Aging Research and Cancer Drug Target, State Key Laboratory of Biotherapy and Cancer Center, National Clinical Research Center for Geriatrics, West China Hospital, Sichuan University, No. 17, Block 3, Southern Renmin Road, Chengdu, Sichuan 610041, PR China.

* Corresponding author: Xiawei Wei, xiaweiwei@scu.edu.cn

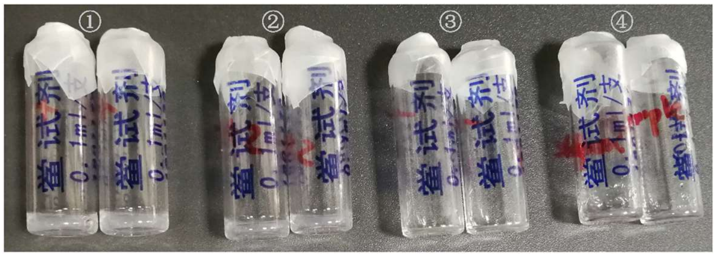

Supplementary figure 1. Endotoxin detection of instillation suspension. The Limulus Amoebocyte Lysate (LAL) gel-clot assay with a sensitivity of 0.25 EU/mL was used. The first two samples are positive controls of endotoxin, the third is negative control, and the fourth is the instillation suspension of CBNPs. After 30 min of incubation, the positive controls reacted with the gel, but the negative control and instillation suspension of CBNPs did not produce a reaction, indicating that the reagent endotoxin levels were less than 0.25 EU/mL.

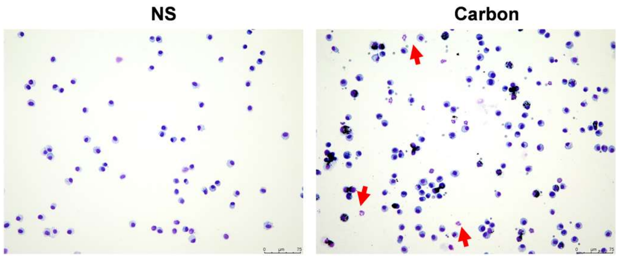

Supplementary figure 2. CBNP-induced neutrophil recruitment into the airways. C57BL/6 mice were instilled with saline containing 0.2 mg of CBNPs. After 24 h exposure, BALF was extracted for cytospin slides**.** Alveolar macrophages engulfed carbon black particles and suffered cell damage, accompanied by remarkable infiltration of neutrophils (Red arrow: neutrophil). Also, a small number of lymphocytes and eosinophils were infiltrated into BALF. Scale bar = 75 μm.

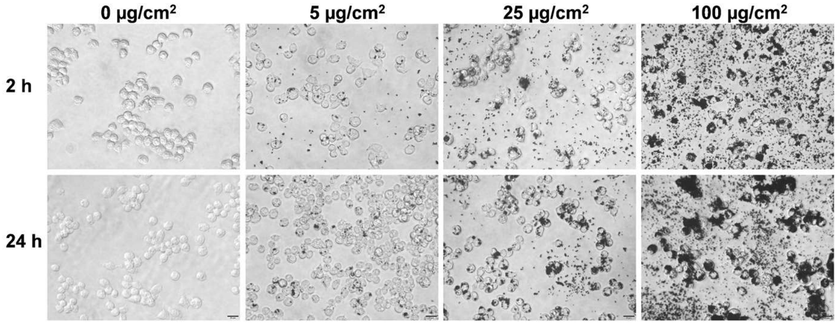

Supplemental figure 3. CBNP-induced morphological changes of macrophages. MH-S cells were treated with CBNPs for 2 h and 24 h at 0, 5, 25 and 100 μg/cm^2. The number of black particle-filled cells increased as exposure time and dose increased. After 2 h of exposure, the particles were located inside and outside the cells. Upon exposure for 24 h, all cells were fully loaded with black particles and were manifested by morphological changes such as cell swelling and cell membrane rupture. Black particles were released following cell lysis and located around necrotic cells. Scale bar = 20 μm.

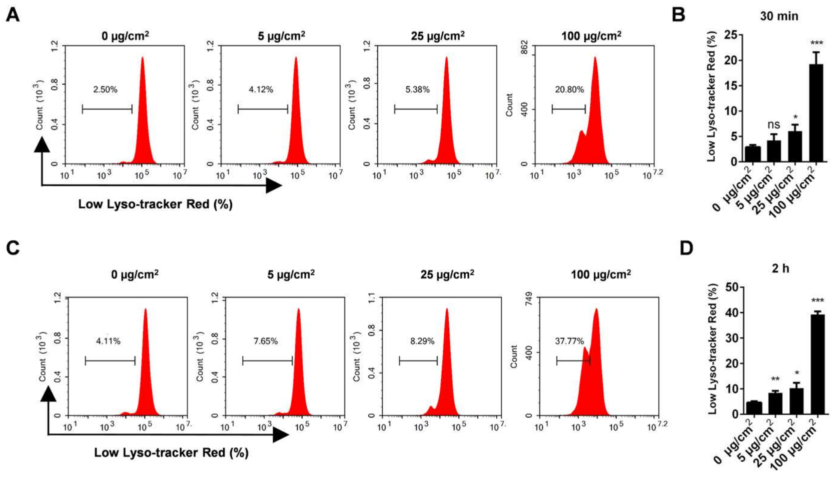

Supplemental figure 4. CBNP-induced lysosomal membrane permeabilization.

(A, C) The fluorescence intensity of lysotracker-red in MH-S cells was detected by flow cytometry after treatment with CBNPs (0-100 μg/cm^2) for 30 min (A) and 2 h (C). (B, D) The percentages of cells in low fluorescence intensity subgroup were compared. Data are mean ± SEM; ns: no significance, *P < 0.05, **P < 0.01, ***P < 0.001 compared with the control group by Student's t-test.

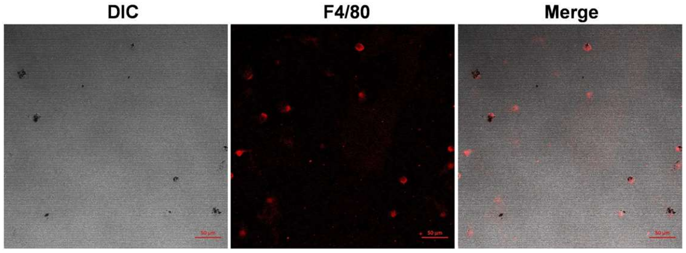

Supplemental figure 5. F4/80 staining of alveolar macrophages. After 24 of CBNP instillation, alveolar macrophages were isolated by adherent culture. The adherent cells were stained by F4/80 (Red) and as observed the cells were positive for F4/80 staining and black particle-filled cells were positive for F4/80 staining. Scale bar = 50 μm.

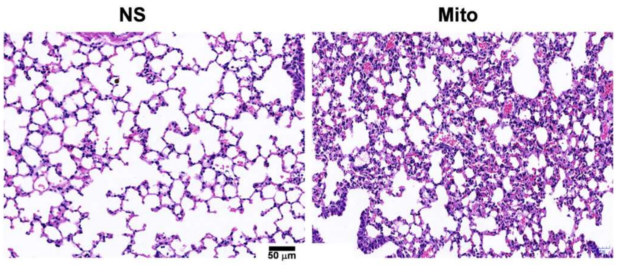

Supplemental figure 6. Pulmonary inflammation triggered by mitochondria from CBNP-treated macrophages. Primary alveolar macrophages were isolated from mice and incubated with CBNPs (100 μg/cm^2) for 4 h. The mitochondria of the cells were extracted and instilled in vivo (200 μg/mouse). Representative mouse lung sections of HE staining 24 h after exposure are presented. Scale bar = 50 μm.

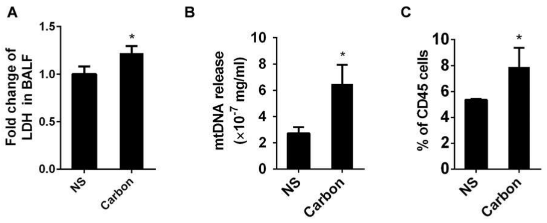

Supplemental figure 7. CBNPs at a low dose induced lung toxicity. The mice with instilled with 0.01 mg of CBNPs. Four hours later, LDH release in BALF was detected (A), and 12 h later, the mtDNA release in BALF was detected (B), and 24 h later, the neutrophils in lung digests were detected (C). *P < 0.05 by Student's t-test.
